# Placental gene-expression profiles of intrahepatic cholestasis of pregnancy reveal involvement of multiple molecular pathways in blood vessel formation and inflammation

**DOI:** 10.1186/1755-8794-7-42

**Published:** 2014-07-07

**Authors:** QiaoLing Du, YouDong Pan, YouHua Zhang, HaiLong Zhang, YaJuan Zheng, Ling Lu, JunLei Wang, Tao Duan, JianFeng Chen

**Affiliations:** 1 Department of Obstetrics, Shanghai First Maternity and Infant Hospital, Tongji University School of Medicine, Shanghai 200040, China; 2 State Key Laboratory of Cell Biology, Institute of Biochemistry and Cell Biology, Shanghai Institutes for Biological Sciences, Chinese Academy of Sciences, Shanghai 200031, China

**Keywords:** Microarray, Intrahepatic cholestasis of pregnancy, Placenta, Genome-wide, Immune response

## Abstract

**Background:**

Intrahepatic cholestasis of pregnancy (ICP) is a pregnancy-associated liver disease with potentially deleterious consequences for the fetus, particularly when maternal serum bile-acid concentration >40 μM. However, the etiology and pathogenesis of ICP remain elusive. To reveal the underlying molecular mechanisms for the association of maternal serum bile-acid level and fetal outcome in ICP patients, DNA microarray was applied to characterize the whole-genome expression profiles of placentas from healthy women and women diagnosed with ICP.

**Methods:**

Thirty pregnant women recruited in this study were categorized evenly into three groups: healthy group; mild ICP, with serum bile-acid concentration ranging from 10–40 μM; and severe ICP, with bile-acid concentration >40 μM. Gene Ontology analysis in combination with construction of gene-interaction and gene co-expression networks were applied to identify the core regulatory genes associated with ICP pathogenesis, which were further validated by quantitative real-time PCR and histological staining.

**Results:**

The core regulatory genes were mainly involved in immune response, VEGF signaling pathway and G-protein-coupled receptor signaling, implying essential roles of immune response, vasculogenesis and angiogenesis in ICP pathogenesis. This implication was supported by the observed aggregated immune-cell infiltration and deficient blood vessel formation in ICP placentas.

**Conclusions:**

Our study provides a system-level insight into the placental gene-expression profiles of women with mild or severe ICP, and reveals multiple molecular pathways in immune response and blood vessel formation that might contribute to ICP pathogenesis.

## Background

Intrahepatic cholestasis of pregnancy (ICP) is the most frequent liver disease arising in the second or third trimester of pregnancy. Clinically, ICP is characterized by maternal pruritus, deranged liver function, elevated serum level of bile acids, and spontaneous relief of signs and symptoms in the mother within 2–3 weeks after delivery [1,2]. The prevalence of this disease has been found to be variable, ranging from 0.1-4.0% [1,3]. In contrast to the benign effects on the mother, ICP has severe consequences for the fetus: it is associated with an increased risk of fetal preterm delivery (19–60% of ICP cases), fetal distress (22–41%), and fetal loss (0.4–1.6%) [4]. However, the etiology and pathogenesis of ICP remain elusive.

An indispensible criterion for ICP is elevation in the serum levels of bile acids, which is considered to be the most appropriate laboratory parameter for diagnosis of the condition [5-7]. During a healthy pregnancy, bile acids are synthesized by the fetus from the 12th week of gestation and exported into the maternal circulation across a steep transplacental gradient [8,9]. In cases of maternal cholestasis, however, the serum levels of bile acids are elevated in both maternal and fetal serum, resulting in a reversal in the transplacental gradient and consequently impairing the export of bile acids from the fetus into the maternal circulation [10,11]. Rates of fetal complication are positively correlated with the total level of bile acids in maternal serum of ICP patients: the probability of fetal complications increases by 1–2% for each additional 1 μM of bile acids, whereas the probability of these events did not increase until bile-acid levels >40 μM [6]. Although the association of maternal serum bile-acid level and fetal outcome has been reported, the underlying mechanisms remain unknown.

The placenta connects the developing fetus to the uterine wall and allows nutrient uptake, waste elimination, and gas exchange between the fetal and maternal blood supplies. The placenta is believed to play a critical role in ICP pathogenesis [12]. Previous studies using human placental tissues or a rodent model of ICP have revealed several morphological abnormalities in ICP placentas, including focally thickened amniotic basement membranes, chorionic villi that are small for gestational age, crowding and congestion of the villi, and an increased number of syncytial knots [12,13]. In addition, increased apoptosis, oxidative stress, and hypoxia have been observed in rat ICP placentas [12]. Thus, in this study, to reveal the molecular mechanisms for the association of maternal serum bile-acid level and fetal outcome in ICP patients, we categorized the ICP patients into two groups (mild ICP, with serum bile-acid concentration ranging from 10–40 μM; severe ICP, with bile-acid concentration >40 μM) and used DNA microarray to characterize the whole-genome expression profiles of placentas. Our system-level analyses of placental gene-expression profiles of ICP, coupled with validation of predicted tissue phenotypes, reveal multiple molecular pathways in immune response and blood vessel formation that might contribute to ICP pathogenesis.

## Methods

### Ethics statement

This study was approved by the Research Committee for Human Subjects, Shanghai Tongji University School of Medicine. All participants provided their written informed consent to participate in this study.

### Patients and tissues

**Figure 1:**
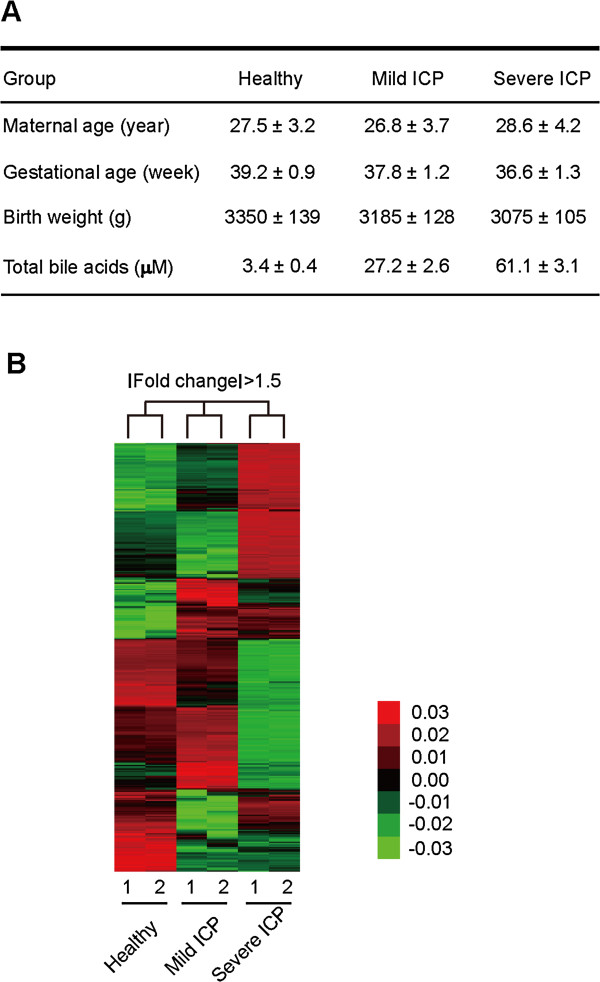
**Hierarchical clustering of differentially expressed genes in placentas from healthy pregnancies and women with ICP. (A)** Clinical features of healthy women and women with mild or severe ICP. 10 pregnant women were recruited for each group, and data are shown as mean ± s.d. (*n* = 10). **(B)** Hierarchical clustering of genes whose expression was altered by more than 1.5-fold in ICP placentas comparing to those in healthy placentas. Red and green represent individual genes that were differentially up-regulated or down-regulated, respectively.

Thirty pregnant women recruited in this study were categorized into three groups (ten pregnancies in each group) according to the serum bile acid level at gestational age of 32 weeks: healthy group with serum bile acid concentration < 10 μM; mild ICP, with serum bile acid concentration ranging from 10–40 μM; and severe ICP, with bile acid concentration >40 μM (Figure 1A). ICP was diagnosed with the following symptoms: 1) presence of classical pruritus with no rash and quick disappearance after delivery; 2) raised maternal serum bile-acid concentration (>10 μM); 3) absence of itching skin disease; and 4) absence of any other causes of liver dysfunction, including preeclampsia; hemolysis, elevated liver enzymes and low platelets (HELLP) syndrome; acute fatty liver of pregnancy; primary biliary cirrhosis; viral hepatitis; and any ultrasound abnormality. All of the ICP patients were not treated with UDCA under their consents. Exclusion criteria for recruitment of healthy pregnant women were similar to that for ICP cases.

To avoid artefact from effects of labor, only placental samples obtained from women who had not undergone labor were selected. All samples were collected immediately after Caesarean sections, cut near the center zone of maternal surface villous lobule after deciduas and amnionic membranes were removed, and then rinsed with saline to remove maternal blood.

### Total serum bile acids analysis

Fasting Serum samples were taken from women and measured twice every week since the gestational age of 32 weeks until caesarean section. Total bile acids were assessed by using an enzymatic method (Kit No Bl 3863, Randox Laboratoires Ltd, United Kingdom) on a Hitachi 7180 biochemistry analyzer (Hitachi, Tokyo, Japan).

### RNA preparation

Freshly isolated placental tissues were divided and dissected separately on ice-cooled RNase-free surfaces. Each sample was placed into an individual tube before addition of 1 mL of TRIzol reagent (Invitrogen), followed by homogenization with a Polytron mixer (Kinematica). Total RNA was extracted according to manufacturer’s instructions (Invitrogen). For DNA microarray, ten pregnant women in each group were randomly divided into two sub-groups and equal amounts of RNA from five individuals in each sub-group were pooled for one array to reduce the variations among different individuals [14,15].

### DNA microarray and hybridization

Agilent Human 4 × 44 K Gene Expression Arrays, which target 27 958 Entrez Gene RNAs, were employed in this study. Sequences were compiled from a broad survey of sources and were subsequently verified and optimized by alignment to the assembled human genome. Coupled with Agilent’s probe selection and robust validation processes, this design delivers increased data quality and less redundancy in gene coverage.

Sample labeling and array hybridization were performed according to the Agilent One-Color Microarray-Based Gene Expression Analysis protocol (Agilent Technology). Briefly, total RNA from each sample was linearly amplified and labeled with Cy3-UTP. The labeled cRNAs were purified using the RNeasy Mini Kit (QIAGEN). The concentration and specific activity of the labeled cRNAs (pmol Cy3/μg cRNA) were measured using a NanoDrop ND-1000. One microgram of each labeled cRNA was fragmented by addition of 11 μl 10× Blocking Agent and 2.2 μl 25× Fragmentation Buffer followed by heating at 60°C for 30 min. Finally, 55 μl 2× GE Hybridization buffer was added to dilute the labeled cRNA. One hundred microliters of hybridization solution was dispensed into the gasket slide, which was assembled with the gene expression microarray slide, followed by incubation for 17 hours at 65°C in an Agilent Hybridization Oven. The hybridized arrays were washed, fixed, and scanned with the Agilent DNA Microarray Scanner (part number G2505B).

### Data analysis and differentially expressed genes sorting

Agilent Feature Extraction software (version 11.0.1.1) was used to analyze acquired array images. Quantile normalization and subsequent data processing were performed using the GeneSpring GX v12.0 software package. Genes that had flags in at least two out of six arrays were chosen for further analysis. Detailed gene expression data are available at the GEO website under accession number GSE46157.

To sort the differentially expressed genes in mild ICP and severe ICP compared with healthy pregnancy, microarray data were analyzed by using linear models and empirical Bayes method, which is considered to be able to generate a reliable statistical inference when the number of arrays is small [16,17]. This method is similar to a standard t test for each probe except that the SES are moderated across genes to get more stable results, which prevents a gene with a very small fold change from being judged as differentially expressed due to an accidentally small residual SD. The resulting *p* values were further adjusted using the BH FDR algorithm [18]. Subsequently, differentially expressed genes were sorted according to fold change (±1.5-fold or greater), followed by secondary selection based on *p*-value and FDR threshold (p < 0.05, FDR < 0.05, controlling the expected FDR to no more than 5%).

### Hierarchical clustering of differentially expressed genes

Hierarchical clustering was performed using the Agilent GeneSpring GX software (version 12.0), and the results were expressed as a dendrogram in which genes and samples with a similar expression pattern formed clusters.

### Gene Ontology (GO) analysis

Gene Ontology (GO) analysis was applied to characterize the primary functions of the differentially expressed genes based on the key functional classification of NCBI [19]. Fisher’s exact test was used to classify the GO category. A *p*-value was calculated for each GO term for all differentially expressed genes and subsequently corrected by false-discovery rate (FDR) [20]. A smaller FDR is associated with a smaller error in judging the *p*-value. Next, enrichment analysis was performed to find GO terms with more specific functions. The enrichment R_e_ was given by R_e_ = (*n*_*f*_/*n*)/(*N*_*f*_/*N*), where *n*_*f*_ is the number of differential genes within the particular category, *n* is the total number of genes within the same category, *N*_*f*_ is the number of differentially expressed genes in the entire microarray, and *N* is the total number of genes on the microarray.

### Gene co-expression network

To identify modules of co-expressed genes [21], we built the gene co-expression network according to the normalized signal intensities of specific genes. We calculated the Pearson’s correlation for each pair of genes and chose those whose values fit the selection criterion to construct the network [22]. Degree of centrality, defined as the number of links between one node and the others, was used as the simplest and most important measure of the centrality of a gene within a network, which in turn determines that gene’s relative importance. In addition, the k-core (from graph theory) was introduced to simplify the graph-topology analysis for the purposes of locating the core regulatory factors (genes) in the gene co-expression network. The k-core of a gene co-expression network usually contains a cohesive group of genes [23-25]. A k-core sub-network with a higher k-core level is considered to have core status within a large-scale gene network.

### Quantitative real-time PCR (qPCR)

Total RNA of each placental sample was prepared as described in RNA preparation. Synthesis of cDNA was carried out using Reverse Transcriptase M-MLV (RNase H-; TaKaRa) and oligo-dT as the primer. QPCR was performed on an Applied Biosystems 7500 Fast Real-Time PCR System using Power SYBR Green PCR Master Mix (Applied Biosystems, Foster City, CA, USA). Primer pairs used were listed in Additional file 1: Table S1. Reactions were performed in triplicate under standard thermocycling conditions (one cycle of 94°C for 4 min, followed by 45 cycles of 94°C for 30 s, 58°C for 30 s, and 72°C for 40 s), and the mean threshold cycle number was used.

### Histology and immunohistochemistry

Freshly isolated placental tissues were fixed with 4% PFA overnight at 4°C, embedded in paraffin, sectioned at 4-μm thickness, and followed by hematoxylin and eosin staining.

Immunohistochemical analyses were performed using the LSAB kit (ZSGB-Bio, Beijing, China). Briefly, paraffin sections were deparaffinized in xylene and rehydrated sequentially in ethanol. The sections were microwaved in EDTA buffer (pH 8.0) to retrieve antigens, incubated in 3% H_2_O_2_ (10 min, room temperature) to inactivate endogenous peroxidase. Sections were incubated with blocking solution (15 min, 37°C), followed by incubation in mouse anti-human CD45 antibody (1:200, 304002, Biolegend) diluted in blocking buffer (overnight, 4°C). The samples were then incubated with biotinylated secondary antibody (15 min, 37°C), followed by incubation with streptavidin conjugated with HRP (15 min, 37°C) and staining with DAB substrate. Nuclei were lightly counterstained with hematoxylin. Finally, slides were dehydrated sequentially in ethanol, cleared with xylenes, and mounted with neutral resin.

For immunofluorescence staining, tissue was fixed in 4% PFA overnight at 4°C, immersed in sucrose solutions, embedded in OCT (Tissue Tek) and sectioned. Frozen sections were incubated with 10% FBS (PBS) to block nonspecific binding sites (1 h, RT) and further incubated with mouse anti-humam CD19 antibody (Alexa Fluor® 700 conjugated, HIB19, 1:200, BD Pharmingen) or mouse anti-human CD3 antibody (FITC conjugated, APA1/1, 1:200, BD Pharmingen) at 4°C overnight. Nuclei were stained with DAPI.

The stained sections were imaged using an Olympus BX51 microscope coupled with an Orca CCD camera (Q-IMAGING).

### Quantifications of blood vessels and immune cells

The HE staining image was used for blood vessel quantification. Ten sections of the terminal villi at the same position in each placenta were examined and 5 random fields in each slide were selected (50 fields total for each placenta) to avoid areas of placental infarction and intervillous fibrin deposition, arterial vessels forming the primary stem and anchoring villi; and histological artifact. The selected fields were then analyzed using ImageJ to count the number of capillaries in each villus. Only terminal villi showing outlines completely within the microscopic field were analyzed. The number of capillaries per villus was calculated by the total number of capillaries divided by the number of terminal villi in 50 fields for each placental sample.

For immune cell quantification, ten sections of the terminal villi at the same position in each placenta were examined and the number of different immune cells in 5 random fields in each section were counted (50 fields total for each placenta). The average of cell number per field was calculated for each placenta.

### Statistical analysis

*P* values of qPCR results, immune cell quantification and blood vessel quantification were calculated by analysis of variance (ANOVA) with Dunnett post-tests. Significant differences are indicated as: *, *p* < 0.05; **, *p* < 0.01. Each experiment was repeated independently for three times.

## Results and discussion

First we employed DNA microarray to identify differentially expressed genes in mild ICP and severe ICP compared to those in healthy pregnancies. Hierarchical clustering of genes whose expression was altered by more than 1.5-fold revealed that gene expression profiles of the two parallel microarrays in each group were highly consistent, indicating the reproducibility of microarray data (Figure 1B). Moreover, as can be observed from the heat map of clustered genes, the three groups of pregnancies had distinctive gene-expression profiles (Figure 1B).

**Figure 2:**
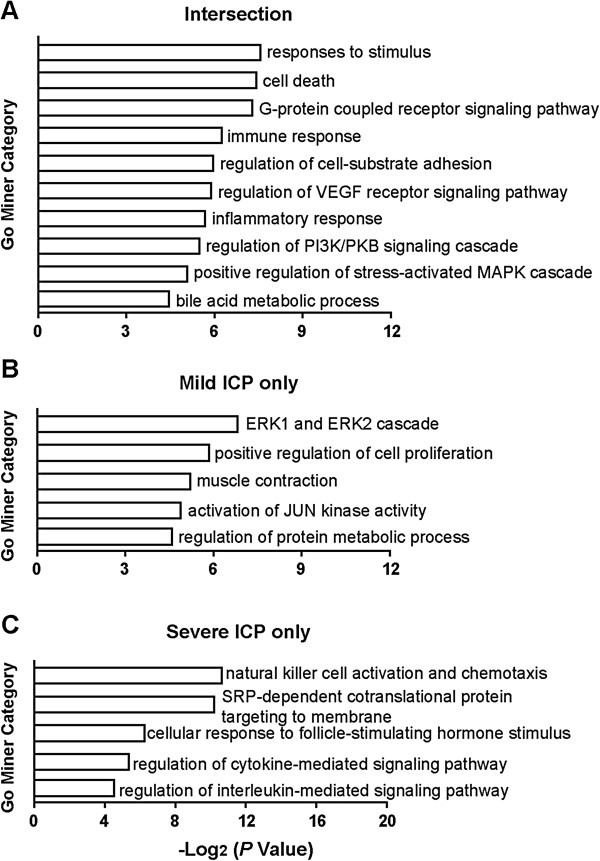
**Significantly enriched Gene Ontology (GO) terms associated with ICP pathogenesis.** The primary functions of the sorted differentially expressed genes in ICP placentas were assessed by GO analysis. Ten significantly enriched GO terms in both mild ICP and severe ICP (Intersection, **A**) and five significantly enriched GO terms in mild ICP only **(B)** and severe ICP only **(C)** are shown respectively. The vertical axis is the GO category and the horizontal axis is the enrichment value of GO.

Subsequently, we applied Gene Ontology (GO) analysis to assess the primary functions of the differentially expressed genes [19] (Figure 2A–C). There was extensive overlap between the GO terms of mild ICP and severe ICP, indicating a strong correlation between mild ICP and severe ICP (Figure 2A). In addition, it is worthwhile to note that the GO terms specifically enriched in severe ICP were mainly related to immune-related signaling pathways (Figure 2C).

**Figure 3:**
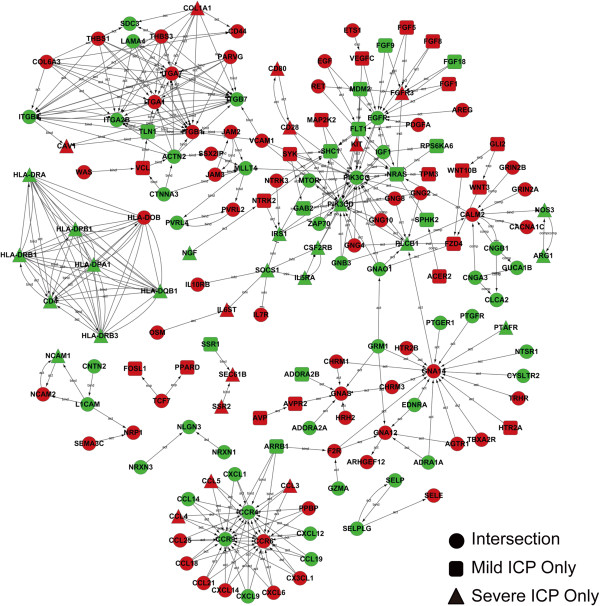
**Gene-interaction network.** Genes associated with the significantly enriched GO terms were analyzed by gene-interaction network, which was built according to the relationships among genes, proteins, and compounds in the KEGG database. Circles, squares, and triangles represent, respectively, genes differentially regulated in both mild ICP and severe ICP (Intersection), mild ICP only, and severe ICP only. Red and green represent genes that were differentially up-regulated or down-regulated, respectively. Detailed gene-gene relationships with the degree of each gene are shown in Additional file 3: Spreadsheet S1.

To further characterize the core regulatory genes involved in the pathogenesis of ICP, genes associated with the significantly enriched GO terms were used to construct gene-interaction and gene co-expression networks [21,26] (Figure 3 and Additional file 2: Figure S1). Detailed gene-gene relationships with the degree of each gene from gene-interaction network and gene co-expression network are shown in Additional file 3: Spreadsheets S1 and Additional file 4: Spreadsheets S2, respectively. These two networks identified the following 19 core regulatory genes: *CCL3*, *CCL25*, *CXCL6*, *CXCL14*, *CCR4*, *CCR6*, *CCR9* and *IL-7R* involved in immune response; *VEGFC*, *FZD4*, *FGF9*, *FGF18*, *ITGB3*, and *FLT1* involved in VEGF signaling pathway; and *GNAS*, *GNA12*, *GNA14*, *EGF*, and *EGFR* involved in G-protein-coupled receptor (GPCR) signaling.

**Figure 4:**
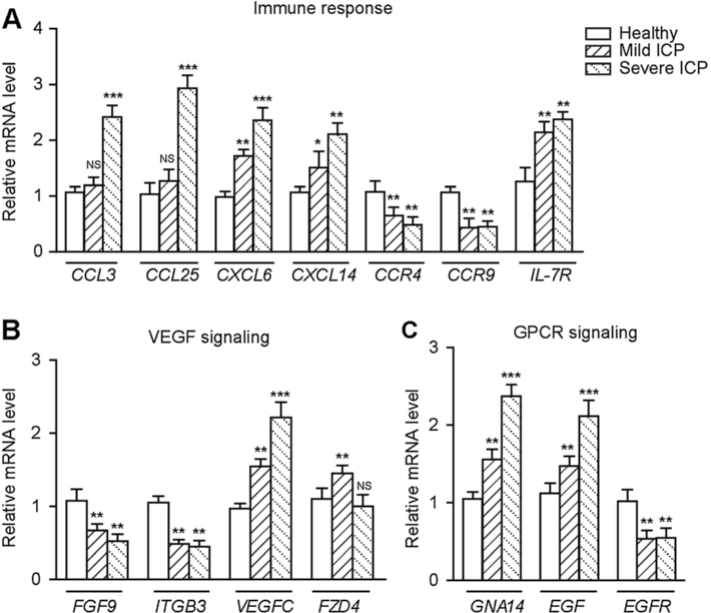
**Validation of the identified core regulatory genes associated with ICP pathogenesis by qPCR.** Genes with expression patterns in placental tissues from healthy group, mild ICP, and severe ICP (10 pregnancies per group) consistent with those in the microarray data are shown: Genes involved in **(A)** immune response, **(B)** VEGF signaling and **(C)** G-protein-coupled receptor (GPCR) signaling. Relative expression of each target gene was normalized to GAPDH, and expression levels in the healthy group were defined as 1. All reactions were performed in triplicate, and data are presented as mean ± s.d. (*n* = 10). *p < 0.05; **p < 0.01; ***p < 0.001; NS, not significant. Primer pairs used were listed in Additional file 1: Table S1.

Next we used qPCR to confirm the expression patterns of the identified 19 core regulatory genes in 30 individuals in all three groups (Additional file 1: Table S1). 14 of 19 detected genes exhibited expression patterns consistent with those from the microarray data, confirming the accuracy of the microarray data (Figure 4).

Despite decades of research, pregnancy is characterized by quite an unusual number of rather frequent conditions with unknown etiology. Although previous studies have provided some information on the altered gene expression profile in ICP placentas [14,27], they did not discriminate between patients with mild ICP and severe ICP. In this study, to further reveal the influence of different bile acid concentration in the pathogenesis of ICP, we sub-grouped the ICP patients into mild and severe ICP according to their serum bile acid level. In addition, as ICP women delivered at earlier gestational stage than healthy pregnant women (Figure 1A), it is possible that different gestational ages of healthy and ICP groups might affect the mRNA expression of placentas. Nevertheless, we were not able to collect placentas from healthy women and women with ICP of the same gestational age in clinical cases, which is a common problem encountered by other studies on the placental gene expression profiles of healthy pregnant women and women with ICP [14,27].

**Figure 5:**
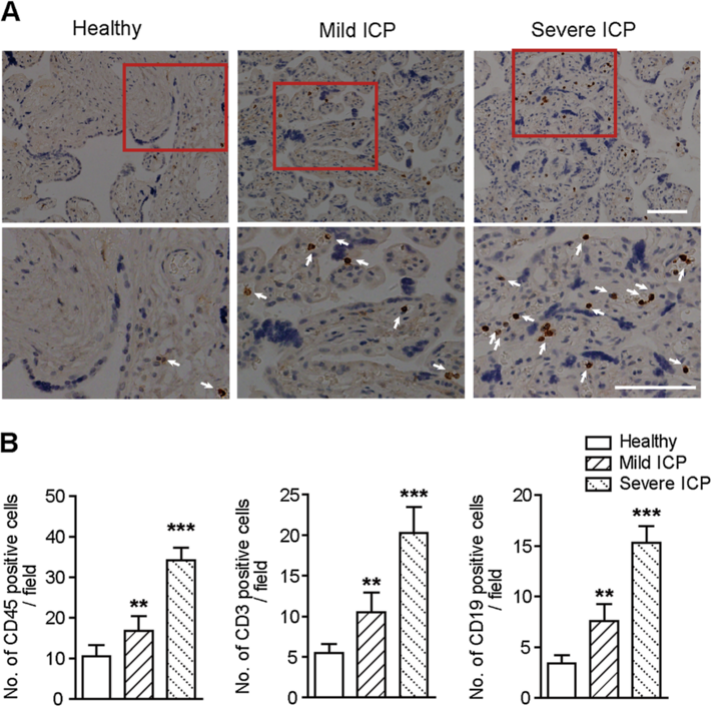
**Immune cell infiltration in placental tissues from healthy group, mild ICP, and severe ICP. (A)** Representative images of immunohistochemistry staining for the leukocyte marker CD45 in placental tissues from healthy group, mild ICP, and severe ICP (10 pregnancies per group). Brown staining (arrows) indicates reactivity. Scale bars represent 100 μm. **(B)** Quantification of leukocytes (CD45 positive), T cell (CD3 positive) and B cell (CD19 positive) in placental tissues from healthy group, mild ICP, and severe ICP (10 pregnancies per group). Data are presented as mean ± s.d. (*n* = 10). **p < 0.01; ***p < 0.001.

Like Gleicher has pointed out in a number of publications, these unexplained conditions of pregnancy exhibit characteristics similar to immunological conditions observed in organ transplant rejection [28] and/or graft versus host disease (GVHD) [29], therefore ICP may represent the complications of normal tolerance of the fetal semi-allograft by the maternal immune system [30]. Herein, our microarray results revealed that genes associated with immune response, *CXCL6*, *CXCL14* and *IL-7R* were up-regulated in mild ICP and further up-regulated in severe ICP, while *CCL3* and *CCL25* were only up-regulated in severe ICP, which suggest that abnormal immune response in patients’ placentas might be positively correlated to the severity of ICP. To explore this implication, we compared the infiltration of CD45 positive leukocytes in placentas from healthy pregnant women and patient with mild or severe ICP. Few leukocytes were present in normal placentas, whereas placentas from mild ICP exhibited more leukocytes aggregation, and placentas from severe ICP displayed massive leukocytes infiltration (Figure 5A). These results are consistent with the gradually elevated expression levels of immune-related genes in mild and severe ICP placentas, and suggest that ICP is associated with immune activation. To further determine which types of immune cells were recruited into placenta in ICP, we stained CD3 positive T cells and CD19 positive B cells in placentas. Compared with normal placentas, placentas from mild ICP showed significantly increased infiltration of both T cells and B cells, and the T/B cells infiltration was further augmented in placenta from patient with severe ICP (Figure 5B). Taken together, our study suggests that ICP is an immunological/inflammatory condition and provides an evidence to support such an interpretation that ICP might be either an allograft rejection mechanism or, more likely, a counterpart to GVHD in organ transplantation.

**Figure 6:**
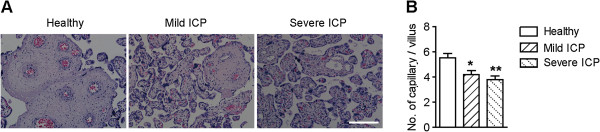
**Histological staining of placental tissues from healthy group, mild ICP, and severe ICP. (A)** Representative hematoxylin and eosin staining of placental tissues from three groups of pregnancies (10 pregnancies per group). Scale bar represents 200 μm. **(B)** Quantification of capillaries per villus of placental tissues from three groups of pregnancies (10 pregnancies per group). Data are presented as mean ± s.d. (*n* = 10). *p < 0.05; **p < 0.01.

Development of a normally functioning placental vascular network to support increasing oxygen and metabolic demands of the growing fetus is necessary for a successful pregnancy. This requires substantial coordination among different vascular endothelial cell-specific growth factors and cell types and is exquisitely dependent on signals exchanged between these cells [31]. As revealed by this study, ICP was accompanied by aggregated immune cell infiltration and gene expression levels of *FGF9* and *ITGB3* were significantly down-regulated in ICP placentas (Figure 5A and Figure 4B). Given the fact that immune cells are able to intimately regulate vessel formation and function [32] and considering the essential role of FGF9 and integrin β3 in vasculogenesis [33] and angiogenesis [34], we postulated that formation and maturation of placental blood vessels might be impaired under conditions of ICP. To test this hypothesis, we subjected placental tissues from healthy and ICP pregnancies to hematoxylin and eosin staining to visualize the vascular structure. Normal placentas exhibited well-organized vascular structure consisting of large vessels (Figure 6A). By contrast, placentas from mild or severe ICP exhibited fewer and smaller blood vessels in each villus (Figure 6A,B), indicating disturbed placental vascular formation and deficient vascular maturation. Interestingly, placental gene expression of *FZD4*, which plays an essential role in vascular development [35], was increased in mild ICP but not in severe ICP (Figure 4B). Since previous studies have shown that *FZD4* expression can be induced by hypoxia [35], it is speculative that up-regulation of *FZD4* in mild ICP may serve as a mechanism to compensate for fluctuations of oxygen and hypoxia that result from lack of vessel formation and blood supply. However, this type of compensation does not exist under condition of severe ICP, which may explain, albeit partially, observations of dilated maternal vascular lacunae in severe ICP placentas (Figure 6A). Moreover, it is worthwhile to note that, gene expression of *VEGFC* was up-regulated in both mild ICP and severe ICP (Figure 4B). Given the function of VEGFC in blood vessel formation [36,37] and the fact that high levels of bile acids exert a constrictive effect on isolated human placental chorionic veins [38], it is tempting to speculate that VEGFC expression was up-regulated as a compensation for hypoxia induced by elevated concentrations of bile acids in ICP placentas.

Additionally, in ICP placentas, we observed differential regulation of critical regulatory genes involved in GPCR signaling, including *EGF*, *EGFR*, and genes encoding G-protein subunits such as *GNA14* (Figure 4C). Because GPCR signaling plays important roles in immune response, this finding implies a potential link between GPCR signaling and the abnormal immune activation in placentas of ICP patients.

## Conclusions

In conclusion, our study provides a system-level insight into the placental gene-expression profiles of women with mild or severe ICP, and reveals multiple molecular pathways in immune response and blood vessel formation that might contribute to ICP pathogenesis. These findings should facilitate elucidation of the mechanisms of ICP development and may suggest therapeutic strategies for management of this disease.

## Abbreviations

ICP: Intrahepatic cholestasis of pregnancy; HELLP: Elevated liver enzymes and low platelets; GO: Gene ontology; FDR: False-discovery rate; qPCR: Quantitative real-time PCR; ANOVA: Analysis of variance; GPCR: G-protein-coupled receptor; GVDH: Graft versus host disease.

## Competing interests

There is no conflict of interest that could be perceived as prejudicing the impartiality of the research reported.

## Authors’ contributions

All authors contributed to the concept, design, acquisition of data or analysis, interpretation of data, drafting or revising the content and approved the final version.

## Authors’ information

QiaoLing Du, M.D., Chief physician, Department of Obstetrics, Shanghai First Maternity and Infant Hospital, Tongji University School of Medicine, China.

YouDong Pan, Ph.D., post-doc, State Key Laboratory of Cell Biology, Institute of Biochemistry and Cell Biology, Shanghai Institutes for Biological Sciences, Chinese Academy of Sciences, China.

YouHua Zhang, Ph.D. candidate, State Key Laboratory of Cell Biology, Institute of Biochemistry and Cell Biology, Shanghai Institutes for Biological Sciences, Chinese Academy of Sciences, China.

HaiLong Zhang, Ph.D. candidate, State Key Laboratory of Cell Biology, Institute of Biochemistry and Cell Biology, Shanghai Institutes for Biological Sciences, Chinese Academy of Sciences, China.

YaJuan Zheng, M.D., technician State Key Laboratory of Cell Biology, Institute of Biochemistry and Cell Biology, Shanghai Institutes for Biological Sciences, Chinese Academy of Sciences, China. 

Ling Lu, Ph.D. candidate, State Key Laboratory of Cell Biology, Institute of Biochemistry and Cell Biology, Shanghai Institutes for Biological Sciences, Chinese Academy of Sciences, China.

JunLei Wang, Ph.D. candidate, State Key Laboratory of Cell Biology, Institute of Biochemistry and Cell Biology, Shanghai Institutes for Biological Sciences, Chinese Academy of Sciences, China.

Tao Duan, M.D., Professor, Director, Department of Obstetrics, Shanghai First Maternity and Infant Hospital, Tongji University School of Medicine, China.

JianFeng Chen, Ph.D., Principle Investigator, Professor, State Key Laboratory of Cell Biology, Institute of Biochemistry and Cell Biology, Shanghai Institutes for Biological Sciences, Chinese Academy of Sciences, China.

## Pre-publication history

The pre-publication history for this paper can be accessed here:

http://www.biomedcentral.com/1755-8794/7/42/prepub

## Supplementary Material

Additional file 1: Table S1 Primer pairs used for quantitative real-time PCR. Click here for file

Additional file 2: Figure S1 Gene co-expression network. Genes associated with the significantly enriched GO terms were analyzed by gene co-expression network using k-core algorithm. Node size represents the strengths of relationships among nodes, and edges between two nodes represent interactions between genes. Genes that connect more edges (i.e., to more genes) play more central roles within the network. Circles, squares, and triangles represent, respectively, genes differentially regulated in both mild ICP and severe ICP (Intersection), mild ICP only, and severe ICP only. Red and green represent genes that were differentially up-regulated or down-regulated, respectively. Detailed gene-gene relationships with degree and k-core of each gene are shown in Additional file 3: Spreadsheet S2. Click here for file

Additional file 3: Spreadsheet S1 The detailed gene-gene relationships in the gene-interaction network. Click here for file

Additional file 4: Spreadsheet S2 The detailed gene-gene relationships from the gene co-expression network. Click here for file
