# Supplementary figures and images for: Placental gene-expression profiles of intrahepatic cholestasis of pregnancy reveal involvement of multiple molecular pathways in blood vessel formation and inflammation
Source: BMC Med Genomics. 2014 Jul 7;7:42. doi: 10.1186/1755-8794-7-42 (PMC4105836; doi:10.1186/1755-8794-7-42)

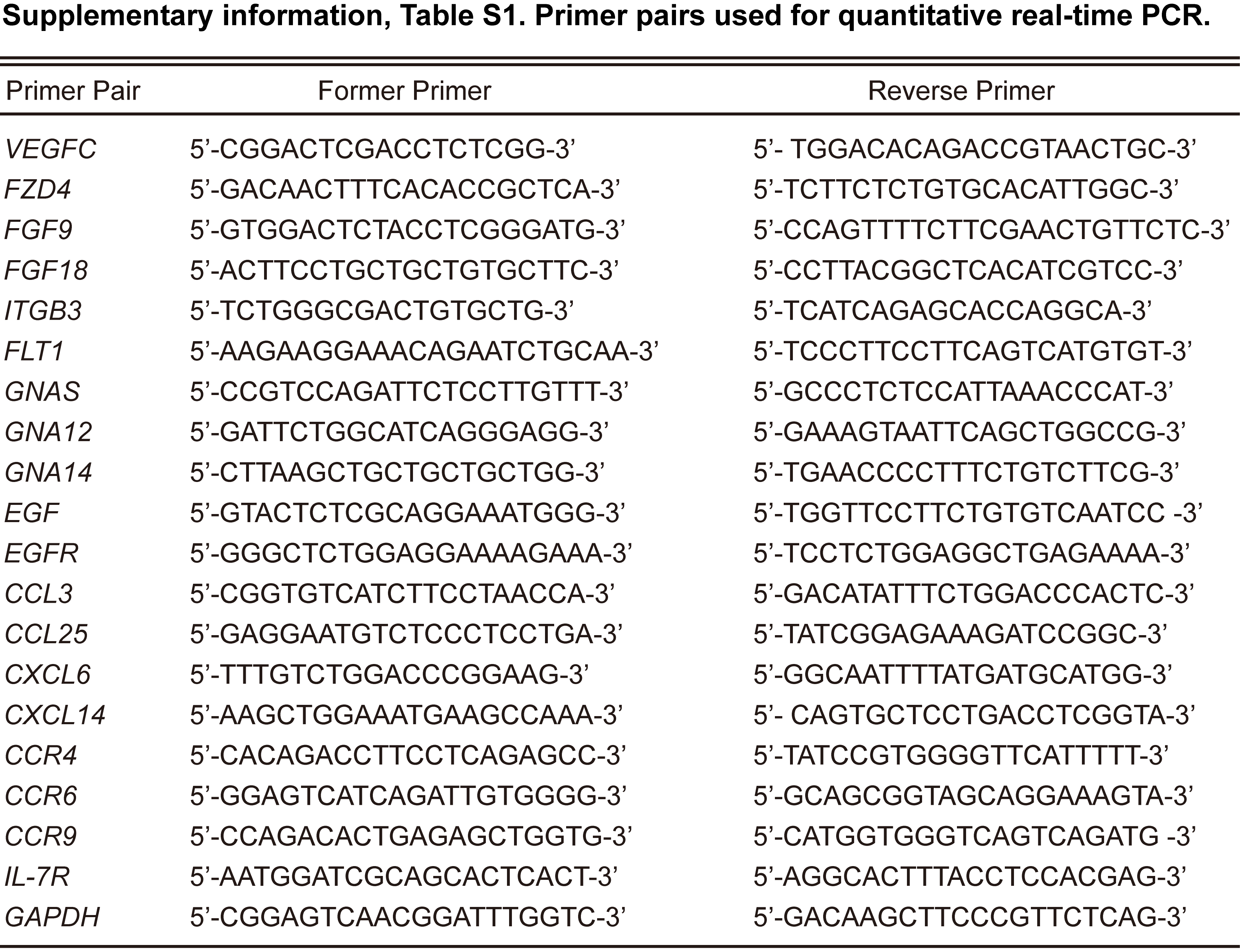

Supplement: Additional file 1: Table S1 — Primer pairs used for quantitative real-time PCR. [file 1755-8794-7-42-S1.tiff]

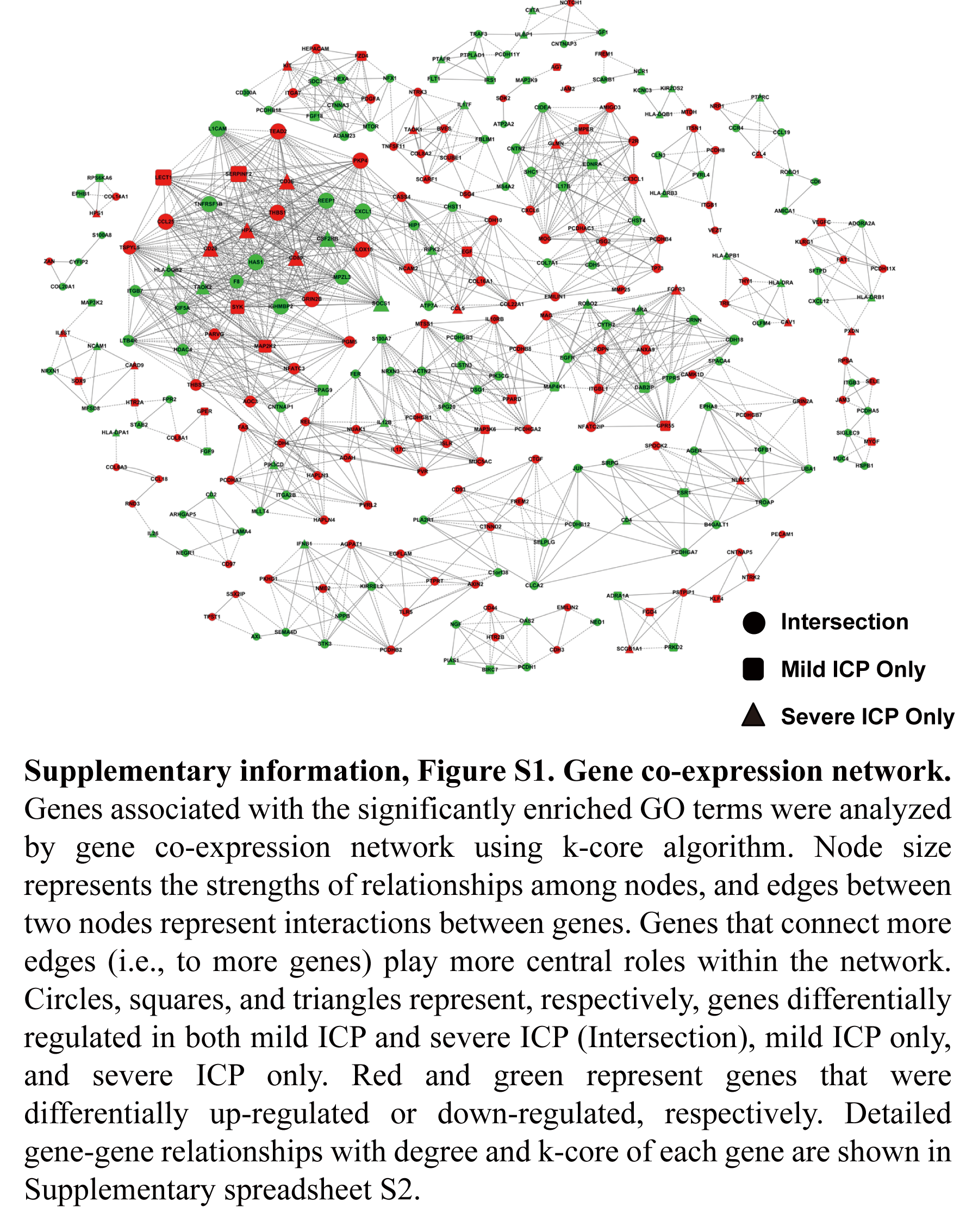

Supplement: Additional file 2: Figure S1 — Gene co-expression network. Genes associated with the significantly enriched GO terms were analyzed by gene co-expression network using k-core algorithm. Node size represents the strengths of relationships among nodes, and edges between two nodes represent interactions between genes. Genes that connect more edges (i.e., to more genes) play more central roles within the network. Circles, squares, and triangles represent, respectively, genes differentially regulated in both mild ICP and severe ICP (Intersection), mild ICP only, and severe ICP only. Red and green represent genes that were differentially up-regulated or down-regulated, respectively. Detailed gene-gene relationships with degree and k-core of each gene are shown in Additional file 3: Spreadsheet S2. [file 1755-8794-7-42-S2.tiff]
